# The effects of livestock grazing on physicochemical properties and bacterial communities of perlite-rich soil

**DOI:** 10.7717/peerj.18433

**Published:** 2024-10-23

**Authors:** Jiraphan Premsuriya, Nontaphat Leerach, Phatcharin Laosena, Woranich Hinthong

**Affiliations:** 1 Princess Srisavangavadhana College of Medicine, Chulabhorn Royal Academy, Bangkok, Thailand; 2 Program in Applied Biological Sciences, Chulabhorn Graduate Institute, Bangkok, Thailand

**Keywords:** Soil metagenomic, Soil physicochemistry, Perlite, Livestock grazing, 16 rRNA gene sequencing

## Abstract

Livestock grazing has been proposed as a cost-effective way to reclaim post-mining lands. It can enhance soil fertility and biodiversity, but its impacts on soil quality and microbial communities vary across soil types. Moreover, waste from grazing raises concerns about pathogens that could pose risks to animal and human health. This study investigated the effects of grazing on post-mining perlite-rich soil in central Thailand. A comparative analysis of soil physicochemical properties and bacterial diversity was conducted between grazed and ungrazed sites. Bacterial diversity was assessed using 16S amplicon sequencing. The perlite-rich soil was found to be sandy, acidic, and to have low nutritional content. Grazing significantly improved the soil texture and nutrient content, suggesting its potential as a cost-effective reclamation strategy. The 16S metagenomic sequencing analysis revealed that microbial communities were impacted by livestock grazing. Specifically, shifts in the dominant bacterial phyla were identified, with increases in Firmicutes and Chloroflexi and a decrease in Actinobacteria. Concerns about increased levels of pathogenic Enterobacteriaceae due to grazing were not substantiated in perlite-rich soil. These bacteria were consistently found at low levels in all soil samples, regardless of livestock grazing. This study also identified a diverse population of Streptomycetaceae, including previously uncharacterized strains/species. This finding could be valuable given that this bacterial family is known for producing antibiotics and other secondary metabolites. However, grazing adversely impacted the abundance and diversity of Streptomycetaceae in this specific soil type. In line with previous research, this study demonstrated that the response of soil microbial communities to grazing varies significantly depending on the soil type, with unique responses appearing to be associated with perlite-rich soil. This emphasizes the importance of soil-specific research in understanding how grazing affects microbial communities. Future research should focus on optimizing grazing practices for perlite-rich soil and characterizing the Streptomycetaceae community for potential antibiotic and secondary metabolite discovery. The obtained findings should ultimately contribute to sustainable post-mining reclamation through livestock grazing and the preservation of valuable microbial resources.

## Introduction

Perlite is an amorphous volcanic rock that has a relatively high water content, is typically formed by the rapid cooling of viscous lava or magma, and mainly consists of aluminosilicate compounds (Reka et al., 2019; Stefanidou, Pachta & Konstantinidis, 2023). Perlite is widely used for many applications, such as in the construction industry, thermal insulators, filtration materials, catalysts, removal of pollutants, and agriculture, given its beneficial physicochemical properties such as low density, high porosity, low thermal conductivity, high heat resistance, chemical inertness, and non-toxicity (Maxim, Niebo & Mcconnell, 2014; Reka et al., 2019; Khoshraftar, Masoumi & Ghaemi, 2023; Yan et al., 2024). Perlite mining has been conducted in many countries, including Greece, China, Iran, Turkey, USA, Japan, Hungary, Italy, Russia, Ukraine, Macedonia, and Thailand (Saisuthichai, 2006; Maxim, Niebo & Mcconnell, 2014; Reka et al., 2019). Perlite is commonly extracted using surface mining techniques such as ripping or blasting, which result in substantial environmental impacts (Maxim, Niebo & Mcconnell, 2014). Post-mining perlite fields typically have low nutrient availability and are often abandoned without any productive use (Stefanidou, Pachta & Konstantinidis, 2023).

Fa-La-Mee Mountain, located in the Lam Narai volcanic field, Lopburi Province, is the only site of perlite mining (Saisuthichai, 2006) that has been commercially operated (between 1992 and 2017) in Thailand. Following the end of the mining concession, the mine was left without any reclamation plan. After being abandoned for 2 years, the flattened parts of the mining area underwent a natural reclamation process and gradually turned into grasslands that created new pastures for livestock grazing. In these areas, farmers introduced livestock for grazing in the form of mixed-breed cattle and Siamese buffaloes. The livestock are herded into the formerly mined areas during the wet season (June–November) and relocated to other areas during the dry season (December–May), which had been ongoing for 2–3 years at the time of this study. Certain areas of the mine remain steep or covered in dry dipterocarp forest and thus inaccessible to livestock. Research has shown that perlite frequently accumulates near the Earth’s surface in regions with perlite mines, so the surface soils in both grazed and ungrazed sites are uniquely rich in crude perlite (Lexa et al., 2021). Although perlite has been mined in various countries, there are limited data on the physicochemical properties and microbial diversity of perlite-rich soil.

Microbial communities play crucial roles in geochemical cycling, soil structure, and soil ecosystem functioning (Banerjee & Van der Heijden, 2023; Dai et al., 2023). Moreover, certain microorganisms in soil such as Actinobacteria, especially *Streptomyces*, are important natural sources of antibiotics and a vast array of natural products (Donald et al., 2022; Alam et al., 2022). Mining activities, particularly the removal of surface topsoil and vegetation, disrupt soil microbial communities, leading to declines in their diversity and function (Rossum et al., 2016; Sansupa et al., 2021; Gabay et al., 2023). At the flatter parts of Fa-La-Mee Mountain where the land has been naturally reclaimed, the introduction of livestock grazing could significantly alter soil physicochemical properties and the composition of microbial communities (Xun et al., 2018; Wu et al., 2022; Mhuireach, Dietz & Gillett, 2022). Previous studies at a range of different locations have shown that light to medium grazing enhanced soil texture and elevated organic carbon, total nitrogen, and available phosphorus (Xun et al., 2018; Lai & Kumar, 2020; Hassan et al., 2023). Grazing was also found to increase both alpha and beta diversity of bacterial communities, reflecting greater richness and evenness of bacterial species (Wu et al., 2022; Mhuireach, Dietz & Gillett, 2022). Other studies have demonstrated that livestock grazing is a potential cost-effective strategy for post-mining reclamation due to its positive effects on soil aggregation, nutrient availability, and the creation of soil ecosystems that support plant growth (Steward, 2006; Teague & Kreuter, 2020). However, several reports have revealed the association between the waste deposited by grazing animals and a rise in antibiotic-resistant Enterobacteriaceae in soil, which can potentially affect human and animal health (Amador et al., 2017; Sharma et al., 2018; Black et al., 2021).

Against this background, we compared the physicochemical properties and bacterial diversity of perlite-rich soil in the Fa-La-Mee Mountain region of central Thailand, focusing on areas that have been impacted or unaffected by livestock grazing.

## Materials & Methods

### Study location, experimental design, and sample collection

**Figure 1:**
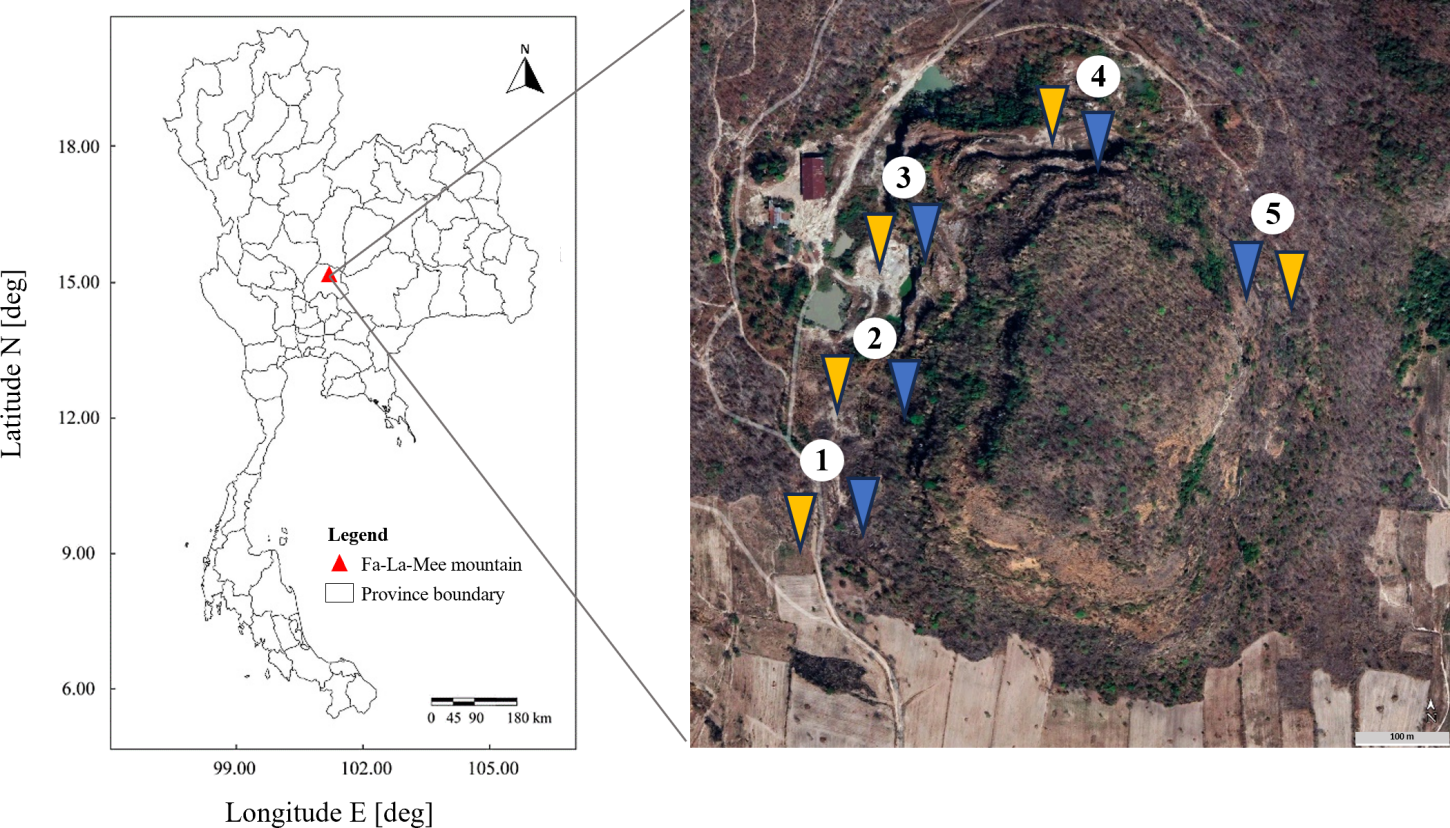
Study areas. Sampling sites are numbered 1–5. Yellow and blue arrows indicate grazed and ungrazed sites, respectively. The aerial image was captured from Google Earth Pro.

This study was performed at an abandoned perlite mine located at Fa-La-Mee Mountain, Lam Narai volcanic field, Lopburi Province, Thailand. Soil samples were collected in December 2022 (dry season) from five different locations approximately 100–200 m apart around the circumference of the mine (Fig. 1). At each location, two sites were designated: one with a history of livestock grazing and one without. This resulted in a total of 10 sampling sites (five grazed and five ungrazed). At each study site, five replicate plots (1 × 1 m) were established. Prior to sample collection, any loose debris on the topsoil was removed. Within each plot, five soil cores were collected from a depth of 10 cm. These cores were then combined to form a composite sample of each plot. The plot-level composite samples were then combined into a single, larger composite sample representing the entire site. The site-level samples were passed through a 2-mm sieve to remove stones, roots, and organic litter. Approximately 1 kg of the combined sample was collected in a sterile container, stored at 4 °C, and transported to the laboratory (method adapted from Sansupa et al., 2021). Three-dimensional representations, coordinates, and descriptions of the grazed and ungrazed sites at the study location are given in Table S1 and Fig. S1.

### Analysis of soil physicochemical properties

Approximately 900 g of soil from each sample site was submitted to the Department of Soil Science, Kasetsart University (Bangkok, Thailand), for analysis of its physicochemical properties. The following variables were measured: pH, texture (sand, silt, clay), electrical conductivity (EC), organic matter (OM), organic carbon (OC), total nitrogen (TN), ammonia (NH_4_), nitrate (NO_3_), available phosphorus (P), available potassium (K), available calcium (C), available magnesium (Mg), available zinc (Zn), available manganese (Mn), available iron (Fe), available sulfate (SO_4_), and soluble silicon (Si). The references for the soil physicochemical property analysis are listed in Table S2.

### DNA extraction and 16S ribosomal RNA (16S rRNA) gene sequencing

To analyze the bacterial composition, 10 g of each soil sample were submitted to Zymo Research (Irvine, CA, USA) for sequencing of the V3–V4 region of the 16S rRNA gene. Soil DNA was extracted using the ZymoBIOMICS DNA Miniprep Kit (Zymo Research). Bacterial 16S rRNA gene targeted sequencing was performed using the Quick-16S™ NGS Library Prep Kit with the Quick-16S™ Primer Set V3–V4 (forward primer 5′-CCTACGGGDGGCWGCAG-3′  and 5′-CCTAYGGGGYGCWGCAG-3′  and reverse primer 5′-GACTACNVGGGTMTCTAATCC-3′; the forward primer is a mixture of the two listed sequences; Zymo Research). The final library was sequenced on Illumina^®^ MiSeq™ with a v3 reagent kit (600 cycles). The sequencing was performed with 10% PhiX spike-in control library (Illumina, San Diego, CA, USA).

### Bioinformatic analysis

Raw DNA reads were processed using QIIME2 (Bolyen et al., 2019). Forward and reverse primers were trimmed, followed by quality filtering, merging of sequences, and chimera removal using DADA2 (Callahan et al., 2016). Subsequently, similar sequences were clustered into amplicon sequence variants (ASVs) at a 100% similarity threshold and into operational taxonomic units (OTUs) at a 97% similarity threshold. ASVs with fewer than 10 sequencing reads were removed. Finally, bacteria were classified taxonomically using the Silva v138 database (Quast et al., 2012). OTUs were employed to examine the overall microbial diversity at the phylum, class, and order levels, while ASVs were used to explore diversity within families and genera.

All bioinformatic analyses were performed in the Galaxy Server (Abueg et al., 2024). Maximum likelihood phylogenetic trees were constructed using MEGA11 (Tamura, Stecher & Kumar, 2021) with the 16S rRNA V3–V4 sequences. The resulting phylogenetic trees were visualized using iTOL (https://itol.embl.de/).

### Statistical analysis

Statistical analyses were performed using PAST software (Hammer et al., 2001) and R program (R Core Team, 2021). The physicochemical properties of the soil were analyzed by non-metric multidimensional scaling (NMDS) using the Bray–Curtis distance. The alpha diversity was calculated based on the observed OTU and diversity indexes (Chao1, Simpson, and Shannon). Beta diversity, reflecting the composition of the bacterial community, was visualized by principal coordinate analysis (PCoA) based on the Bray–Curtis distance of OTU composition. Spearman’s rank correlation was used to determine the effect of soil physicochemical properties on the abundance of bacterial phyla. All of these analyses were performed using PAST software. OTUs with significant differences in abundance among different groups (logarithmic score >2 and *p* < 0.05) were identified by linear discriminant analysis effect size (LEfSe) (Segata et al., 2011). This analysis was performed using the LEfSe tool available on the Huttenhower Lab Galaxy Server (https://huttenhower.sph.harvard.edu/lefse/). LEfSe leverages the relative abundance of amplicon ASVs in each sample, identifies taxa with statistically significant differences between groups using the Kruskal–Wallis test, and estimates their effect sizes through linear discriminant analysis. The sign test was performed for comparing the soil physicochemical properties or relative abundance of bacterial taxa between grazed and ungrazed. Additionally, a paired *t*-test was also employed to assess differences in soil physicochemical properties between these two types of sites. Both the sign test and paired *t*-test were performed using R.

## Results

### Soil physicochemistry

**Figure 2:**
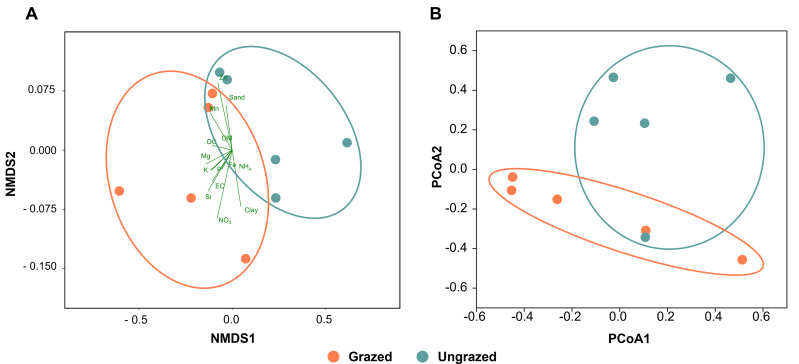
Comparison of the grazed and ungrazed sites regarding soil physicochemical properties and bacterial communities. (A) Non-metric multidimensional scaling of the soil physicochemistry analyzed using the Bray–Curtis distance. (B) Principal coordinate analysis was performed using the Bray–Curtis distance based on operational taxonomic unit composition.

All soil samples appeared sandy with a white, light gray, or gray color, indicating the presence of crude perlite (Fig. S2). Physicochemical analyses revealed that the perlite-rich soils across both grazed and ungrazed sites had sandy texture, acidic pH, and low nutrient content. High variability in most measured parameters across the study sites necessitated the use of both the paired *t*-test and sign test to assess the significance of differences in these parameters between grazed and ungrazed sites. Soils grazed by livestock exhibited significantly higher levels of clay content, EC, OM, OC, TN, NH_4_, NO_3_, available P, available K, available Ca, available Mn, extractable Mn, extractable Fe, extractable SO_4_, and soluble Si compared to ungrazed soils (Table 1). Samples with a history or no history of livestock grazing were clustered separately when analyzed using NMDS (Fig. 2A). The raw physiochemical data on the soils are summarized in Table S3.

**Table 1:** Physicochemical properties of perlite-rich soil.

Parameter	Area		*t*-test		Sign test	
	Grazed	Ungrazed	*t*	*p*-value	*Z*	*p*-value
pH	6.1 ± 0.5	5.5 ± 0.7	2.648	0.057	1.342	0.180
Sand (%)	60.2 ± 17.8	68 ± 15.9	−3.814	0.019	2.236	0.025
Silt (%)	30.4 ± 16.3	23.5 ± 9.1	1.458	0.219	1.342	0.180
Clay (%)	9.6 ± 5.4	6.8 ± 3.9	2.997	0.040	2.236	0.025
EC (dS/m)	0.06 ± 0.03	0.03 ± 0.01	1.835	0.140	2.236	0.025
OM (g/kg)	29.4 ± 23.8	12.7 ± 9.5	1.700	0.164	2.236	0.025
OC (g/kg)	17 ± 13.8	7.4 ± 5.5	1.703	0.164	2.236	0.025
TN (g/kg)	1.3 ± 1.1	0.6 ± 0.4	1.680	0.168	2.236	0.025
NH_4_ (mg/kg)	6.7 ± 4.1	3.5 ± 2.7	1.906	0.129	2.236	0.025
NO_3_ (mg/kg)	8.4 ± 4.1	3.0 ± 2.3	3.023	0.039	2.236	0.025
C/N ratio	11.3 ± 2	12.6 ± 3.4	−0.856	0.417	0.447	0.655
P (mg/kg)	306.2 ± 111.4	135.5 ± 58.4	3.336	0.029	2.236	0.025
K (mg/kg)	35.5 ± 51.5	5.3 ± 2.8	1.325	0.256	2.236	0.025
Ca (mg/kg)	959 ± 606	370.8 ± 226.2	2.397	0.075	2.236	0.025
Mg (mg/kg)	215.6 ± 164	63.6 ± 26.8	2.387	0.075	2.236	0.025
Zn (mg/kg)	1.1 ± 0.6	0.5 ± 0.4	1.247	0.280	1.342	0.180
Mn (mg/kg)	43 ± 21.7	23.6 ± 16.8	3.038	0.038	2.236	0.025
Fe (mg/kg)	54.1 ± 36.9	27.3 ± 25.7	1.771	0.151	2.236	0.025
SO_4_ (mg/kg)	49.1 ± 20.8	38.6 ± 8.5	1.243	0.282	1.342	0.180
Si (mg/kg)	20.5 ± 14.8	8.6 ± 1.7	1.835	0.140	2.236	0.025

### Differences in bacterial composition between grazed and ungrazed sites

**Figure 3:**
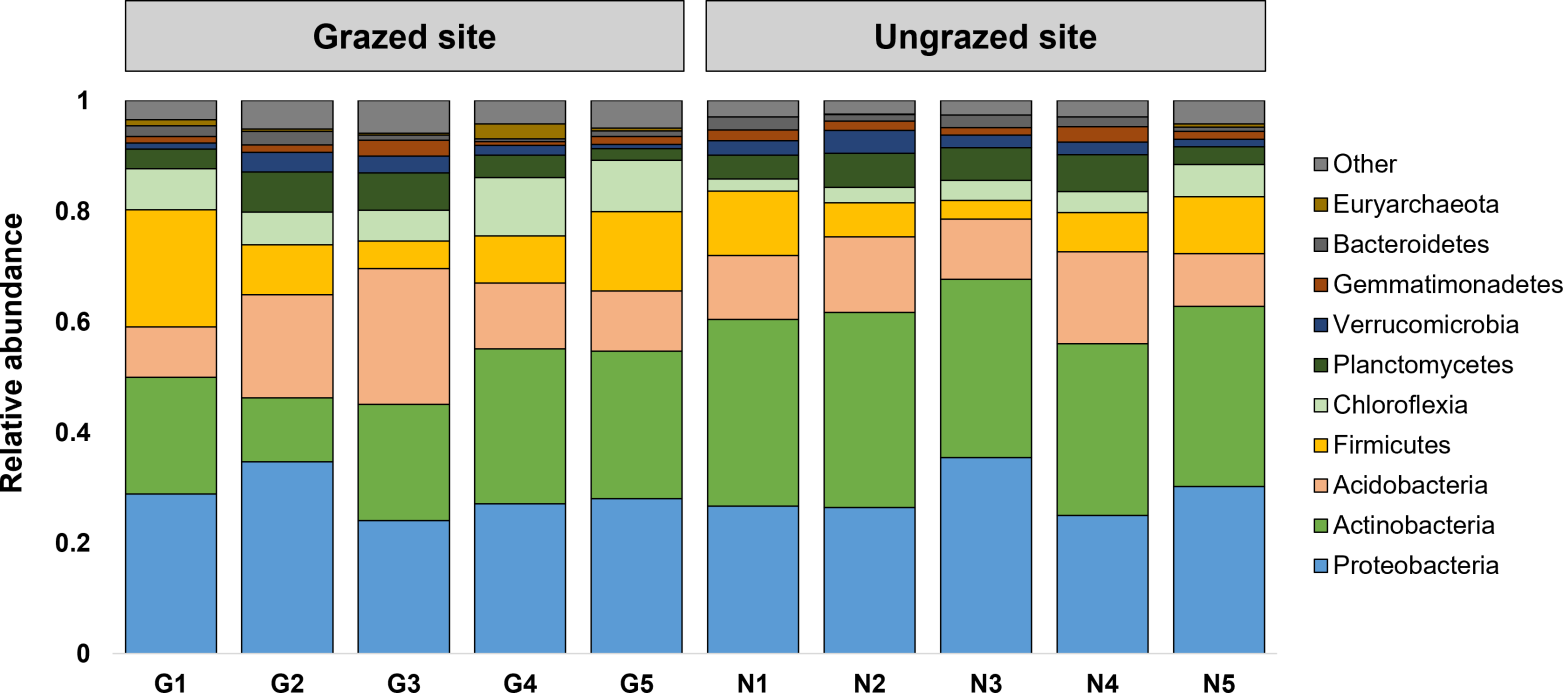
Relative abundance of the 10 most abundant phyla. The stacked bar chart illustrates the relative abundance of the 10 most abundant phyla. Phyla with lower relative abundance are combined in the “Other” category.

**Figure 4:**
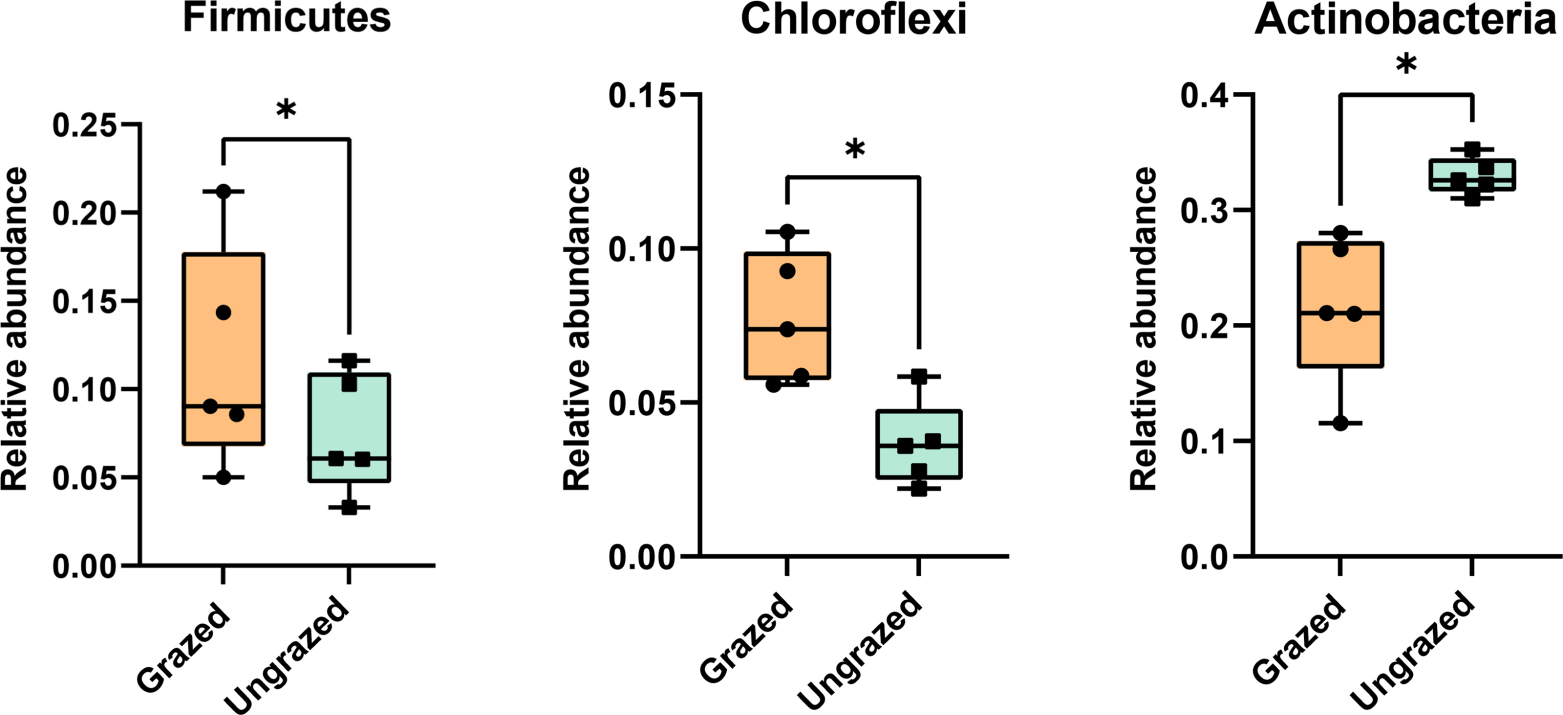
Relative abundance of Firmicutes, Chloroflexi, and Actinobacteria in the different sites. Significance was determined using the sign test (*p* < 0.05).

**Figure 5:**
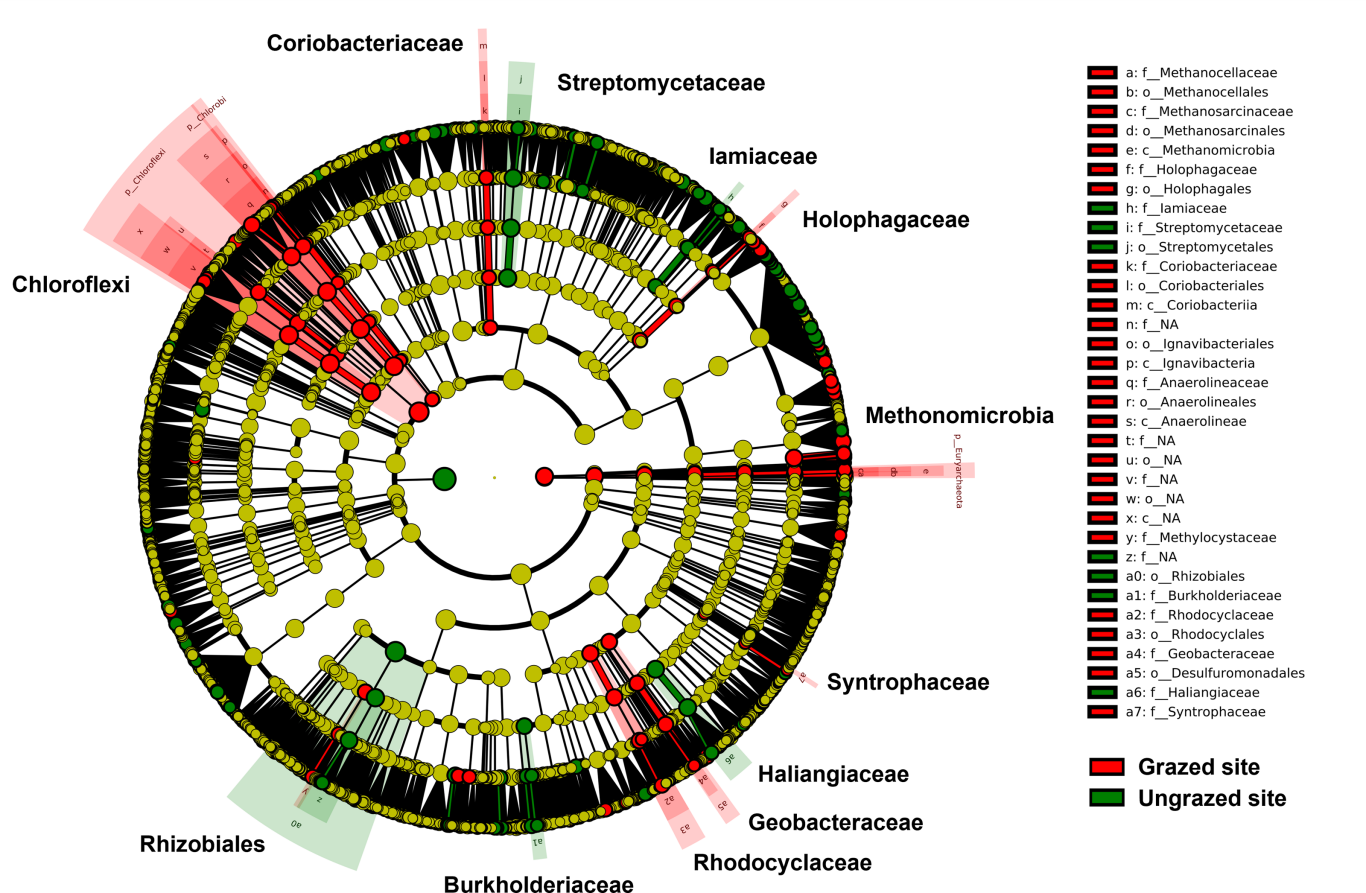
Linear discriminant analysis effect size analysis on operational taxonomic units identified in perlite-rich soil samples from grazed and ungrazed sites. The cladogram shows the differences in enriched taxa in grazed sites (red) *versus* enriched taxa in ungrazed sites (green). Differences among classes were analyzed using the Kruskal–Wallis test.

Rarefaction curves of the observed OTUs from each sample plateaued at the analyzed sequencing depth (20,000 reads per sample), indicating that a sufficient number of OTUs had been detected to represent the bacterial communities as captured by our method (Fig. S3). Alpha-diversity analysis of the observed OTUs revealed no significant differences in biodiversity between the grazed and ungrazed sites (Fig. S4). However, despite the similarities in biodiversity summary statistics, beta-diversity analysis revealed differences in the microbial communities between the grazed and ungrazed sites (Fig. 2B). According to the OTU annotation results, a total of 6,314 OTUs, belonging to 30 phyla, 70 classes, 139 orders, 262 families, and 790 genera, were identified across all samples. The most abundant phyla in perlite-rich soil included Proteobacteria, Actinobacteria, Acidobacteria, Firmicutes, and Chloroflexi. There were distinct differences in the relative abundance of dominant bacterial phyla, especially Actinobacteria, Firmicutes, and Chloroflexia, between sites with and without livestock grazing (Fig. 3). Further analysis also showed that samples exposed to livestock grazing exhibited significant increases in Firmicutes (11.6 ± 0.06% grazed *vs.* 7.6 ± 0.03% ungrazed) and Chloroflexi (7.7 ±  0.02% grazed *vs.* 3.6 ± 0.01% ungrazed), but a significant decrease in Actinobacteria (21.7 ± 0.06% grazed *vs.* 33.0 ± 0.16% ungrazed) (Fig. 4). LEfSe analysis was used to identify taxa that differed significantly between the sample groups (*p* < 0.05 and linear discriminant analysis (LDA) score >2). Samples from areas grazed by livestock exhibited significantly higher abundances of Chloroflexi, Methonomicrobia, Coriobacteriaceae, Holophagaceae, Syntrophaceae, Geobacteraceae, and Rhodocyclaceae, while ungrazed sites exhibited significantly higher abundances of Rhizobiales, Streptomycetaceae, Burkholderiaceae, Iamiaceae, and Haliangiaceae (Fig. 5).

### Abundance and biodiversity of Enterobacteriaceae and Streptomycetaceae in grazed and ungrazed sites

**Figure 6:**
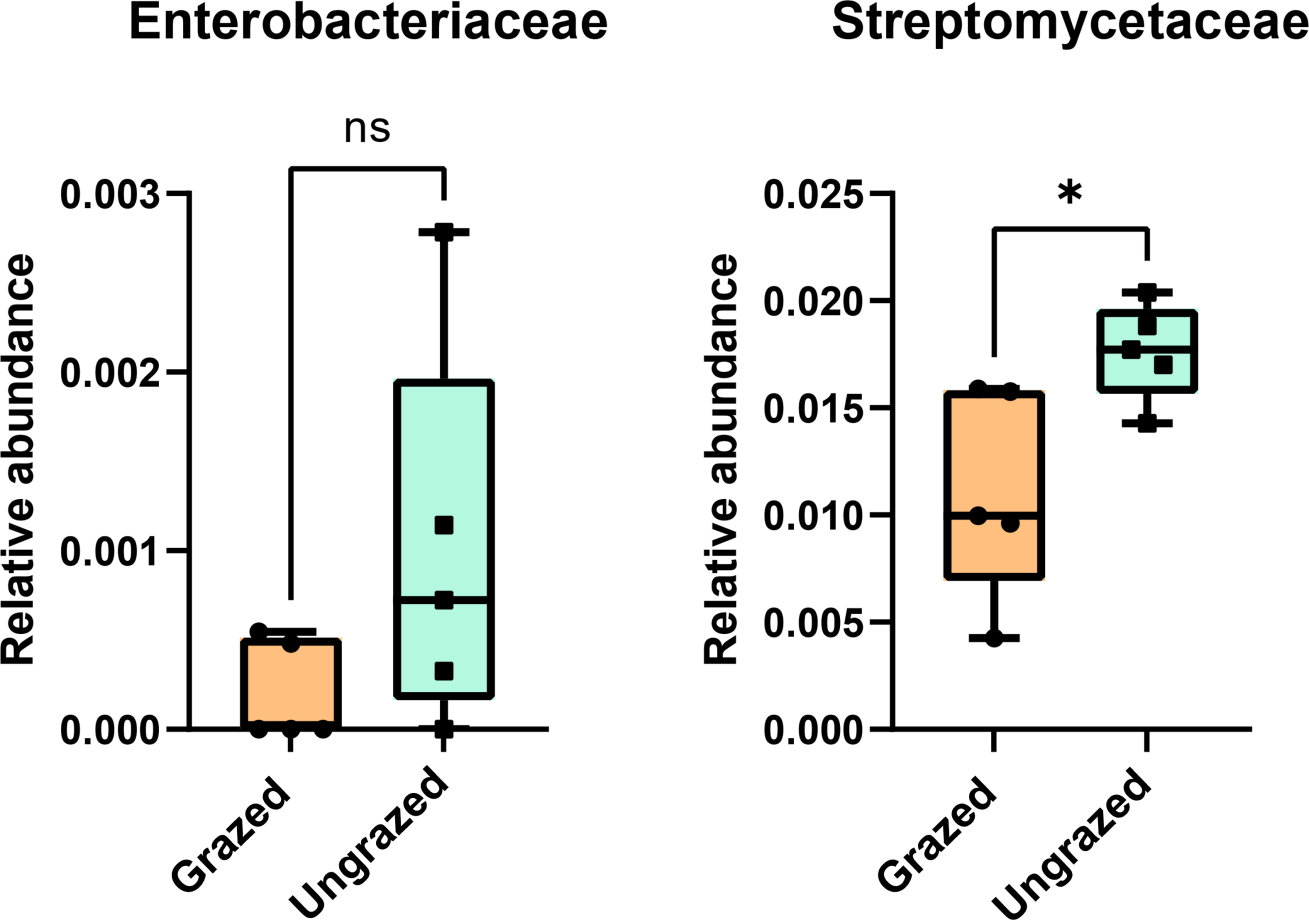
Relative abundance of Enterobacteriaceae and Streptomycetaceae in the different sites. Significance was determined using the sign test (*p* < 0.05).

**Figure 7:**
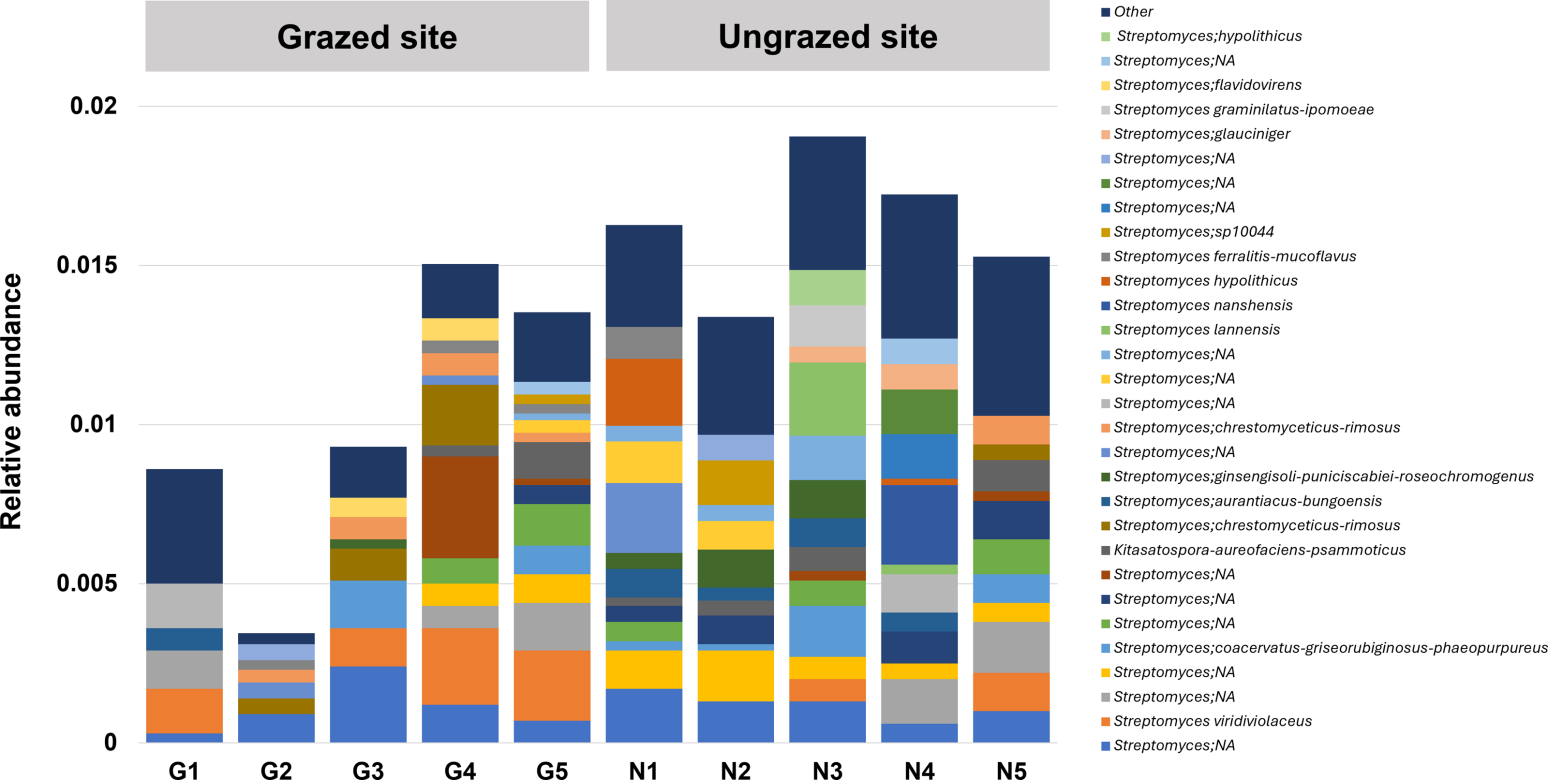
Abundance bar plot of Streptomycetaceae found in perlite-rich soil grazed or ungrazed by livestock. The 30 most abundant taxa are shown individually, while all remaining taxa are grouped together as “Other”.

**Figure 8:**
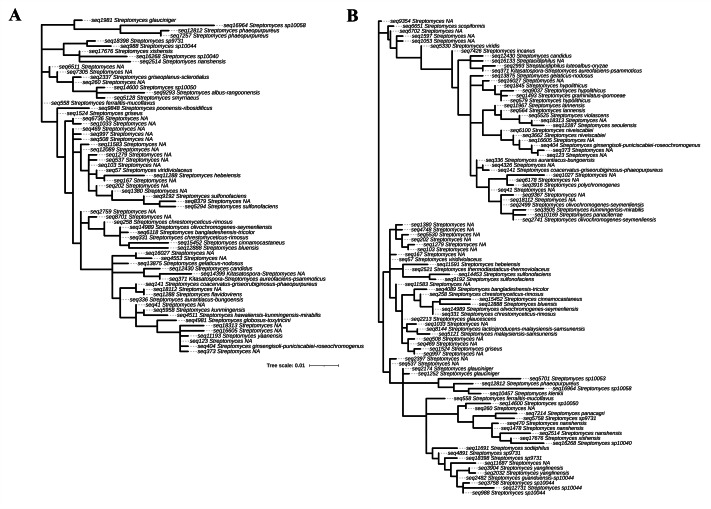
Phylogenetic analysis of Streptomycetaceae found in perlite-rich soil grazed (A) and ungrazed by livestock (B). The phylogenetic tree was constructed based on 16S rRNA (V3–V4) sequences by the maximum-likelihood method using MEGA11.

In this study, 16S rRNA metagenomic sequencing revealed a relatively low abundance (0–0.1%) of Enterobacteriaceae in the perlite-rich soil, with no significant differences between samples with or without a history of grazing (Fig. 6). Interestingly, LEfSe analysis revealed significant differences in the abundance of Streptomycetaceae between grazed and ungrazed sites. This prompted a more detailed analysis of the abundance and biodiversity of this bacterial family. The ungrazed sites had a significantly higher abundance of Streptomycetaceae than the grazed ones (1.76 ± 0.23% and 1.11 ± 0.49%, respectively) (Fig. 6). Regarding biodiversity, 16S rRNA metagenomic sequencing identified 122 ASVs belonging to the Streptomycetaceae family. Of these, 115 ASVs were assigned to the *Streptomyces* genus, while 4 and 2 ASVs were mapped to *Kitasatospora* and *Streptacidiphilus*, respectively. One ASV remained unclassified at the genus level. Interestingly, the ungrazed sites harbored a higher diversity of Streptomycetaceae with 90 ASVs, compared with 60 ASVs found in the grazed sites, while only 28 ASVs were found at both locations. The abundance bar plot visualizes the overall abundance and diversity of Streptomycetaceae of samples from grazed and ungrazed sites (Fig. 7). Phylogenetic analysis of 16S rRNA gene sequences (V3–V4 region) revealed a high degree of diversity among Streptomycetaceae populations within the perlite-rich soil, as evidenced by their segregation into distinct clades within the phylogenetic tree (Fig. 8).

### Relationship between bacterial composition and soil physicochemical properties

**Figure 9:**
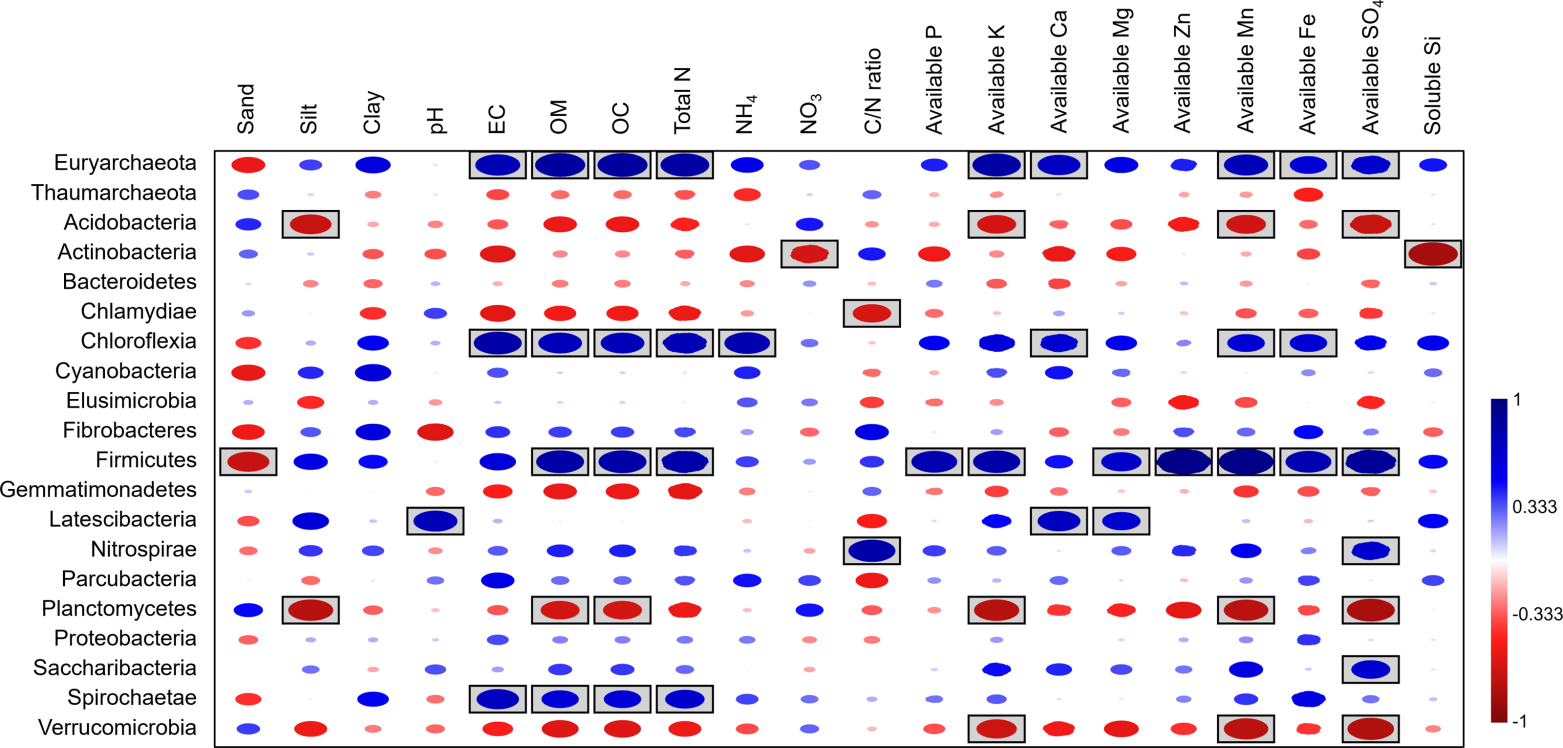
Spearman’s rank correlation between the abundant phyla and soil physicochemical properties. A circle within a box indicates a significant correlation (*p* < 0.05).

Spearman’s rank correlation analysis between bacterial composition at the phylum level and soil physicochemical properties revealed significant positive correlations between the relative abundances of Firmicutes, Chloroflexi, and soil OM, along with several nutrients (*e.g.*, Fe, Mn). In contrast, available K, Mn, and SO_4_ showed negative correlations with the abundance of Acidobacteria, Planctomycetes, and Verrucomicrobia. Additionally, NO_3_ and soluble silicon had negative correlations with Actinobacteria (Fig. 9).

## Discussion

Perlite is a volcanic aluminosilicate mineral that is used in various applications. It has been mined in many countries around the world; once mining ends, the land is in need of reclamation. In this study, we comparatively analyzed the physicochemical properties and bacterial community structure of perlite-rich soil from grazed and ungrazed land surrounding an abandoned perlite mine in central Thailand. This work aims to provide a deeper understanding of how livestock grazing influences the characteristics of perlite-rich soil and how it might help or hinder efforts to reclaim the land for uses such as cropland and forestry.

Physicochemical analysis of perlite-rich soil indicated that it had a sandy texture, acidic pH, and limited nutrient availability. Interestingly, livestock grazing for approximately 2–3 years significantly improved the soil texture and nutrient availability. This aligns with previous studies on other soil types that have demonstrated that rational grazing had positive effects on soil aggregation and nutrient content, while helping the overall development of ecosystems to support plant growth (De Faccio Carvalho et al., 2010; Teague & Kreuter, 2020). Livestock grazing can be a cost-effective strategy for reclaiming post-mining lands (Steward, 2006). However, it has also been reported that uncontrolled or intensive grazing can adversely impact the quality of soil and ecosystem (Lai & Kumar, 2020). Thus, there is an urgent need for further research to identify suitable grazing practices to promote the long-term sustainability and ecological resilience of land reclaimed after the cessation of mining activities.

Microbial communities are keystone components of soil ecosystems, influencing crucial biogeochemical cycles, soil structure development, and overall ecosystem function (Xun et al., 2018; Wu et al., 2022; Mhuireach, Dietz & Gillett, 2022). In this study, 16S rRNA gene sequencing indicated that Proteobacteria, Actinobacteria, Acidobacteria, Firmicutes, and Chloroflexi were the most abundant bacterial phyla in perlite-rich soil, which aligns with findings from previous microbiome studies on various soil types (Xun et al., 2018; Wang et al., 2021; Cui et al., 2021). The results of this study also build on these findings by revealing the potential effects of livestock grazing on the bacterial community in perlite-rich soil. Soil samples from grazed sites revealed significant increases in the relative abundance of Firmicutes and Chloroflexi and a decrease in Actinobacteria. Previous research has demonstrated that livestock grazing impacts soil microbial communities differently depending on the soil type. For example, a study on meadow steppe soils in Inner Mongolia, China identified an increase in Firmicutes and a decrease in Chloroflexi after grazing by cattle for six years (Xun et al., 2018). In contrast, a study on chestnut soil in Inner Mongolia found a significant increase in Chloroflexi abundance with cattle grazing (Zhang et al., 2023). Meanwhile, chernozem (black soil) in Inner Mongolia had decreased Firmicutes and Actinobacteria populations after grazing (Wang et al., 2021), while typical steppe soils in northwest China exhibited elevations in both Firmicutes and Actinobacteria following grazing (Usman et al., 2024). Additionally, a study on desert soil in northwest China showed increases in all three of these phyla (Firmicutes, Chloroflexi, and Actinobacteria) following grazing (Cui et al., 2021). These diverse findings strongly suggest that the effect of livestock grazing on soil microbial communities is highly dependent on the soil type and likely influenced by other variables such as the specific grazing practices employed and grazing duration. Many studies have suggested that the deposition of manure by grazing animals alters the physicochemical characteristics of soil, which can subsequently impact the composition of microbial communities (Xun et al., 2018; Zhang et al., 2023; Usman et al., 2024). Our findings align with this, as the abundance of Firmicutes/Chloroflexi was found to correlate with the levels of soil OC and other nutrients (*e.g.*, Mg, Fe) in grazed sites. Additionally, several studies have shown an association between high abundance of Firmicutes and elevated levels of soil OC and nutrients. This reflects the copiotrophic nature (rapid growth when resources are highly abundant) of Firmicutes and its capacity to decompose recalcitrant carbon sources (Xun et al., 2018; Cui et al., 2021; Lourenço, Cantarella & Kuramae, 2023). However, the responses of Chloroflexi to OC and soil nutrients appear to depend on the soil type (Trivedi et al., 2016; Xun et al., 2018; Cui et al., 2021; Lourenço, Cantarella & Kuramae, 2023). Firmicutes species have been widely studied for their multifaceted contributions to bioremediation and sustainable agriculture. These beneficial bacteria promote plant growth by enhancing nutrient acquisition, influencing the production of hormones by plants, and acting as biological control agents against plant pathogens (Aguilar-Paredes et al., 2023). The phylum Chloroflexi is a diverse group of organisms, including photosynthetic and decomposer species. Despite being one of the most dominant taxa in soils, the ecological significance of Chloroflexi remains poorly understood due to limited data (Thiel et al., 2019).

Several studies have linked the manure deposited by grazing livestock to an increase in antibiotic-resistant Enterobacteriaceae in soil, which poses a potential threat to human and animal health (Amador et al., 2017; Sharma et al., 2018; Black et al., 2021). However, in this study, 16S rRNA metagenomic sequencing revealed a relatively low abundance (0–0.1%) of Enterobacteriaceae in the perlite-rich soil, with no significant difference observed between the grazed and ungrazed samples. A previous study also demonstrated that the survival of *Escherichia coli*, a member of the Enterobacteriaceae family, varied with the soil type but was minimal in sandy soil (Alegbeleye & Sant’Ana, 2023). Another study revealed a strong correlation between soil microbial communities, which differ across soil types, and the survival of Enterobacteriaceae in soil (Moynihan et al., 2015). Therefore, the low abundance of Enterobacteriaceae observed in the perlite-rich soil, regardless of grazing, is likely a consequence of both the physicochemical properties of this soil type and the distinct microbial communities within it.

This study also revealed a high abundance and diversity of Streptomycetaceae within the perlite-rich soil. This is likely attributable to the established capacity of this bacterial family to form biofilms on perlite granules (Domínguez-González et al., 2022). Streptomycetaceae are well recognized for their production of vast numbers and varieties of antibiotic compounds. Furthermore, members of this family can produce numerous secondary metabolites with antifungal, antiparasitic, chemotherapeutic, and immunosuppressant applications. Thus, Streptomycetaceae bacteria are considered a valuable resource for novel antibiotic discovery and other beneficial applications (Donald et al., 2022; Alam et al., 2022). Analysis of the Streptomycetaceae family in perlite-rich soil revealed the presence of three genera: *Streptomyces*, *Kitasatospora*, and *Streptacidiphilus*. Notably, *Streptomyces* was the most abundant genus within this family. *Streptomyces* is the largest antibiotic-producing genus of Actinomycetota, which can produce a vast array of medically important antibiotics, including streptomycin, tetracycline, and chloramphenicol (Donald et al., 2022). *Kitasatospora* have emerged as valuable sources of bioactive compounds due to their ability to produce a variety of biologically active compounds with extremely diverse structures, most of which are unique to this genus (Zimmermann et al., 2023). Finally, the genus *Streptacidiphilus* represents a group of acidophilic members of Actinobacteria within the family Streptomycetaceae (Song et al., 2018). Genome mining studies have identified *Streptacidiphilus* strains as intriguing candidates for further investigation due to their unique enzymatic activities and potential production of novel secondary metabolites (Malik, Kim & Kim, 2020). Interestingly, analysis of the Streptomycetaceae community in the perlite-rich soil revealed the presence of several ASVs that did not match any identified members of Streptomycetaceae within the Silva v138 database. This suggests the possibility that novel, uncharacterized Streptomycetaceae species are present in the study area. This, in turn, highlights that Fa-La-Mee Mountain could be the location of valuable Actinomyces capable of producing novel antibiotics and secondary metabolites. While livestock grazing can enhance soil quality, it adversely affects Streptomycetaceae populations within perlite-rich soils. Specifically, LEfSe analysis revealed a significantly higher abundance of Streptomycetaceae in ungrazed sites than in grazed ones. This was further supported by a significant difference in Streptomycetaceae abundance detected using a sign test. Notably, livestock grazing also affected the overall diversity of Streptomycetaceae in perlite-rich soil. These results differ from observations in paddock and chernozem soil types, for which studies have reported that the abundance and diversity of Streptomycetaceae populations were promoted by livestock grazing (Wang et al., 2021; Mhuireach, Dietz & Gillett, 2022). This discrepancy highlights that the relationship between Streptomycetaceae and grazing practices may be dependent on the soil type.

Taking these findings together, this study and the existing literature emphasize the need for soil-specific research to understand how livestock grazing affects soil microbial communities. This knowledge is crucial for optimizing grazing practices to promote healthy soil ecosystems as well as for conserving microbial resources in different soil environments.

## Conclusions

This study investigated the impact of livestock grazing on the physicochemical properties and microbial communities of perlite-rich soil surrounding an abandoned perlite mine in central Thailand. Our findings revealed that livestock grazing improved soil texture and nutrient availability without increasing the abundance of pathogenic Enterobacteriaceae. This study also revealed a highly abundant and diverse Streptomycetaceae community within the perlite-rich soil. This, in turn, suggests that this environment is potentially a location from which novel antibiotic- and secondary metabolite-producing strains can be isolated. However, livestock grazing led to significant decreases in the abundance and diversity of Streptomycetaceae in the study area. Therefore, further studies on the optimization of grazing practices and the characterization of Streptomycetaceae in perlite-rich soil are required to improve the soil for post-mining reclamation and microbial resource conservation. Our findings, along with the results from previous studies, suggest that the effects of livestock grazing on soil microbial communities vary depending on the soil type. This highlights the critical need for regionally specific soil investigations to develop optimized grazing practices across diverse environments.

##  Supplemental Information

10.7717/peerj.18433/supp-1 Supplemental Information 1 Description of the study area

10.7717/peerj.18433/supp-2 Supplemental Information 2 Reference for soil physicochemical property analysis

10.7717/peerj.18433/supp-3 Supplemental Information 3 Soil physiochemistry raw data

10.7717/peerj.18433/supp-4 Supplemental Information 4 Three-dimensional representation of the sampling sites Yellow and blue arrows indicate grazed and ungrazed sites, respectively. The images were captured from Google Earth Pro.

10.7717/peerj.18433/supp-5 Supplemental Information 5 Textures of the soil samples The samples were photographed before sieving.

10.7717/peerj.18433/supp-6 Supplemental Information 6 Sample-based rarefaction curves of observed bacterial operational taxonomic units for soil samples Rarefaction curves were generated to illustrate the relationship between the number of sequences and the number of observed operational taxonomic units in each sample. Orange lines represent the samples from grazed sites and teal lines represent the samples from ungrazed sites.

10.7717/peerj.18433/supp-7 Supplemental Information 7 Alpha diversity of samples (A) Observed species, (B) Chao1 index, (C) Shannon index, and (D) Simpson index. Significance was determined using sign test (*p* < 0.05).

## Additional Information and Declarations

### Data Availability

The following information was supplied regarding data availability: The raw sequencing data is available at the NCBI SRA: PRJNA1139768.
