# Supplementary material for: The effects of livestock grazing on physicochemical properties and bacterial communities of perlite-rich soil
Source: PeerJ. 2024 Oct 23;12:e18433. doi: 10.7717/peerj.18433 (PMC11512551; doi:10.7717/peerj.18433)
Supplement: Supplemental Information 1 [file peerj-12-18433-s001.docx]

| Area | Coordinates | Area description | |
| --- | --- | --- | --- |
|  |  | Grazed site | Ungrazed site |
| 1 | 15.289291, 100.876245 | Plain, open grassland  Livestock: mixed-breed cattle | Slope covered by dry dipterocarp forest |
| 2 | 15.290831, 100.876875 | Plain, open grassland  Livestock: Siamese buffalos | Ridge areas |
| 3 | 15.292203, 100.877317 | Plain, open grassland  Livestock: mixed-breed cattle | Ridge areas |
| 4 | 15.293443, 100.879061 | Plain, open grassland  Livestock: mixed-breed cattle | Ridge areas |
| 5 | 15.292420, 100.881079 | Plain, open grassland  Livestock: mixed-breed cattle | Slope covered by dry dipterocarp forest |
