# Supplementary material for: The effects of livestock grazing on physicochemical properties and bacterial communities of perlite-rich soil
Source: PeerJ. 2024 Oct 23;12:e18433. doi: 10.7717/peerj.18433 (PMC11512551; doi:10.7717/peerj.18433)
Supplement: Supplemental Information 2 [file peerj-12-18433-s002.docx]

| Soil parameter | Reference |
| --- | --- |
| pH | Thomas, 1996 |
| Texture | Gee & Bauder, 1979 |
| Electrical conductivity | Rhoades, 1996 |
| Organic matter | Walkley & Black, 1934 |
| Organic carbon | Nelson & Sommers, 1996 |
| Total nitrogen | Bremner, 1996 |
| Ammonia | Mulvaney, 1996 |
| Nitrate | Mulvaney, 1996 |
| Available phosphorus | Bray & Kurtz, 1945 |
| Available potassium | Helmke & Sparks, 1996 |
| Available calcium | Loeppert & Suarez, 1996 |
| Available magnesium | Loeppert & Suarez, 1996 |
| Available zinc | Lindsayn & Norvell, 1978 |
| Available manganese | Lindsayn & Norvell, 1978 |
| Available iron | Lindsayn & Norvell, 1978 |
| Available sulphate | Walker, 1972 |
| Soluble silicon | Miller, 1967 |

Bray RH, Kurtz LT. 1945. Determination of Total Organic and Available Forms of Phosphorus in Soils. *Soil Science*, 59, 39-45. DOI:10.1097/00010694-194501000-00006.

Bremner JM. 1996. Nitrogen-Total. In: Sparks, D.L., Ed., Methods of Soil Analysis: Part 3—Chemical Methods, Book Series No. 5, Soil Science Society of America and American Society of Agronomy, Madison, WI, USA.

Gee GW, Bauder JW. 1979. Particle Size Analysis by Hydrometer: A Simplified Method for Routine Textural Analysis and a Sensitivity Test of Measurement Parameters. *Soil Science Society of America Journal*, 43, 1004-1007. DOI:10.2136/sssaj1979.03615995004300050038x.

Helmke PA, Sparks DL. 1996. Lithium, Sodium, Potassium, Rubidium, and Cesium. In: Sparks, D.L., Ed., Methods of Soil Analysis: Part 3—Chemical Methods, Book Series No. 5, Soil Science Society of America and American Society of Agronomy, Madison, WI, USA.

Lindsay WL, Norvell WA. 1978. Development of a DTPA Soil Test for Zinc, Iron, Manganese, and Copper. *Soil Science Society of America Journal.* 42(3), 421-428. DOI: 10.2136/sssaj1978.03615995004200030009x

Loeppert RH, Suarez DL. 1996. Carbonate and Gypsum. In: Sparks, D.L., Ed., Methods of Soil Analysis: Part 3—Chemical Methods, Book Series No. 5, Soil Science Society of America and American Society of Agronomy, Madison, WI, USA.

Miller RW. 1967. Soluble Silica in Soil. *Soil Chemistry*. 31: 46-50. DOI: 10.2136/sssaj1967.03615995003100010016x

Mulvaney RL. Nitrogen—Inorganic Forms. In: Sparks, D.L., Ed., Methods of Soil Analysis: Part 3—Chemical Methods, Book Series No. 5, Soil Science Society of America and American Society of Agronomy, Madison, WI, USA.

Nelson DW, Sommers LE. 1996. Total Carbon, Organic Carbon, and Organic Matter. In: Sparks, D.L., Ed., Methods of Soil Analysis: Part 3—Chemical Methods, Book Series No. 5, Soil Science Society of America and American Society of Agronomy, Madison, WI, USA.

Rhoades JD. Salinity: Electrical Conductivity and Total Dissolved Solids. In: Sparks, D.L., Ed., Methods of Soil Analysis: Part 3—Chemical Methods, Book Series No. 5, Soil Science Society of America and American Society of Agronomy, Madison, WI, USA.

Thomas GW. 1996. Soil pH and Soil Acidity. In: Sparks, D.L., Ed., Methods of Soil Analysis: Part 3—Chemical Methods, Book Series No. 5, Soil Science Society of America and American Society of Agronomy, Madison, WI, USA.

Walker DR. 1972. Soil Sulfate I. Extraction and Measurement. *Canadian Journal of Soil Science*. 52: 253-260.

Walkley AJ, Black IA. 1934. Estimation of soil organic carbon by the chromic acid titration method. *Soil Science*. 37, 29–38.
