# Supplementary figures and images for: The effects of livestock grazing on physicochemical properties and bacterial communities of perlite-rich soil
Source: PeerJ. 2024 Oct 23;12:e18433. doi: 10.7717/peerj.18433 (PMC11512551; doi:10.7717/peerj.18433)

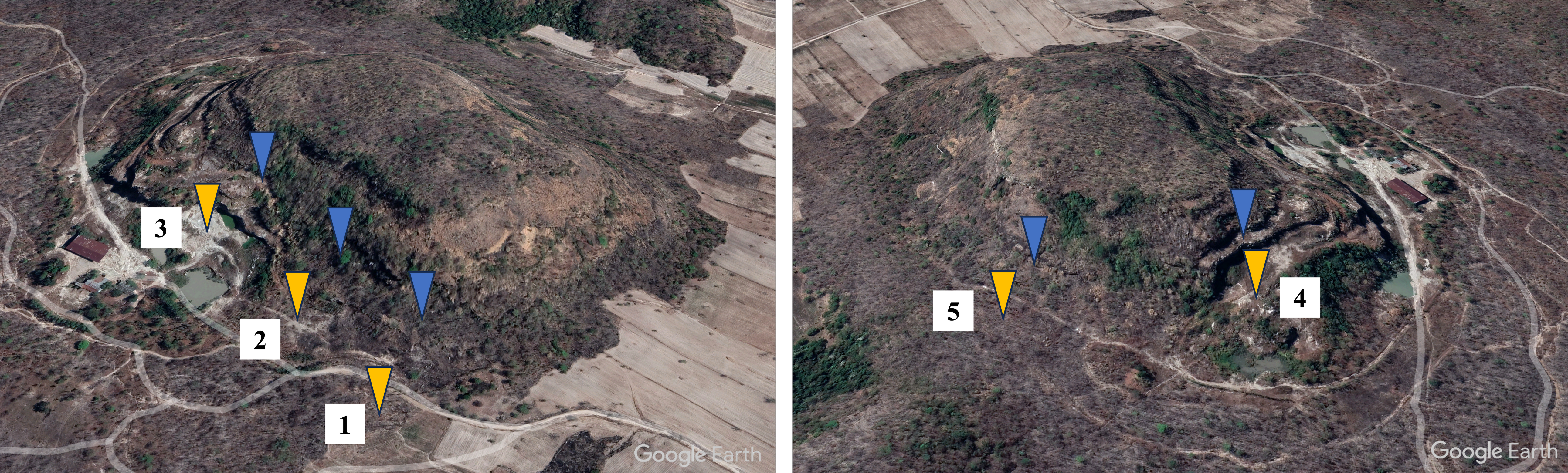

Supplement: Supplemental Information 4 — Yellow and blue arrows indicate grazed and ungrazed sites, respectively. The images were captured from Google Earth Pro. [file peerj-12-18433-s004.png]

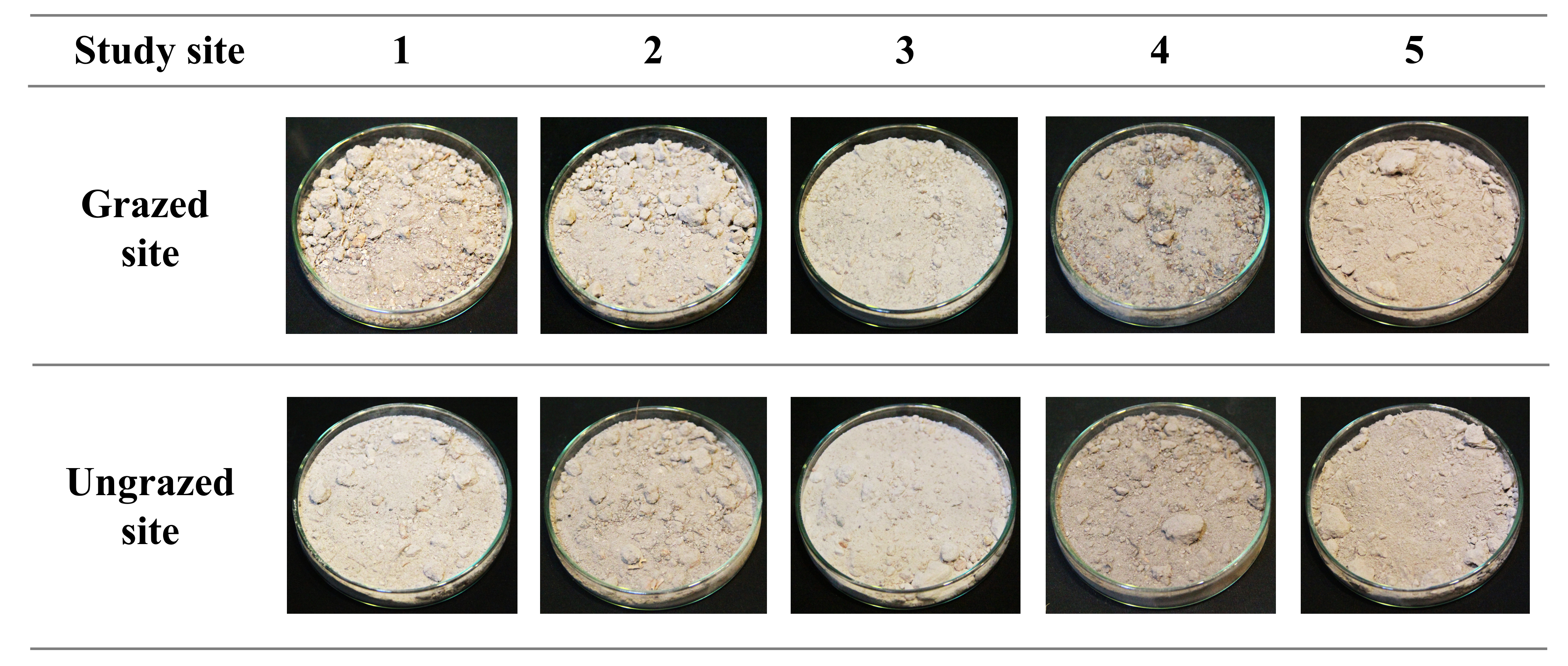

Supplement: Supplemental Information 5 — The samples were photographed before sieving. [file peerj-12-18433-s005.png]

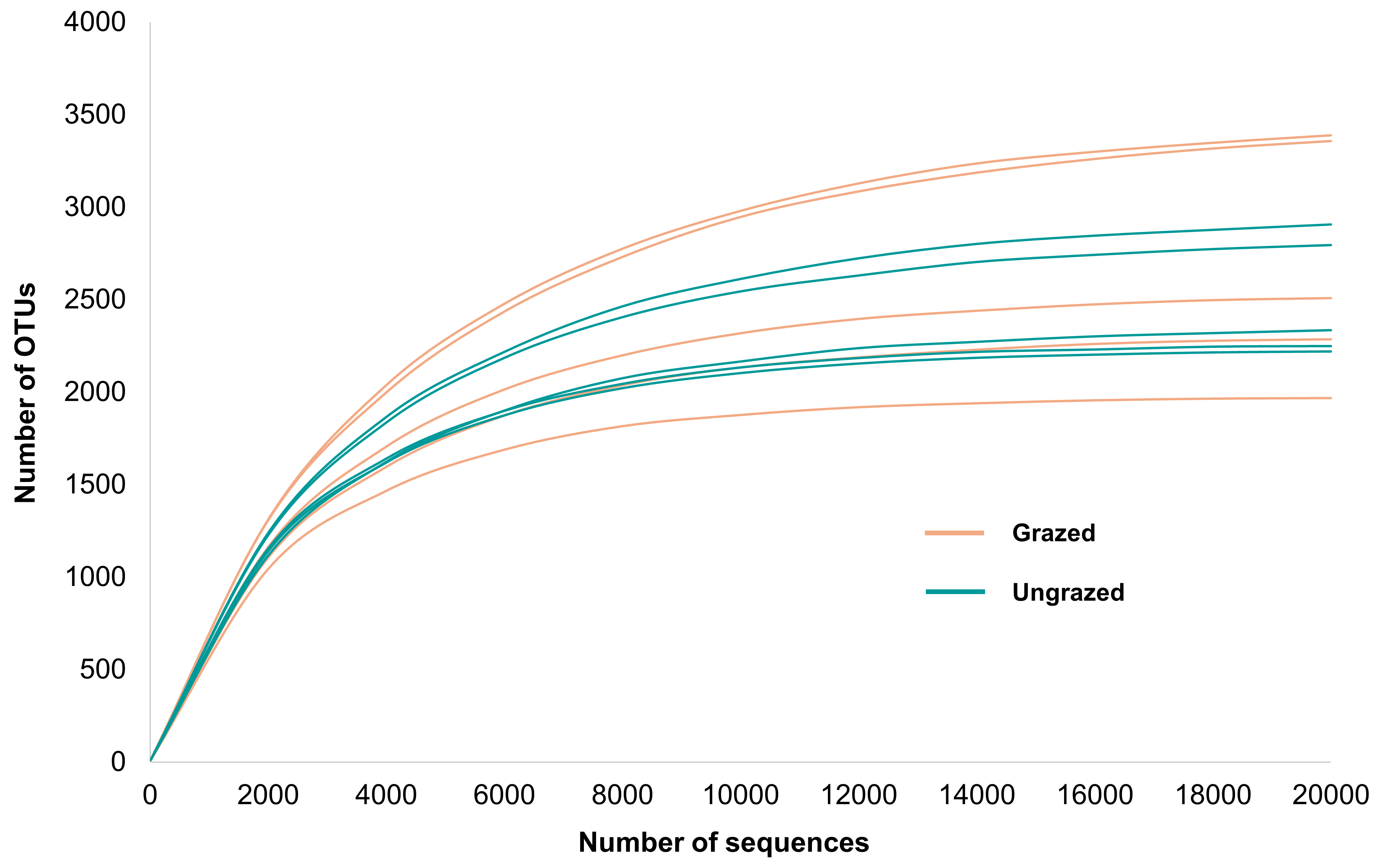

Supplement: Supplemental Information 6 — Rarefaction curves were generated to illustrate the relationship between the number of sequences and the number of observed operational taxonomic units in each sample. Orange lines represent the samples from grazed sites and teal lines represent the samples from ungrazed sites. [file peerj-12-18433-s006.png]

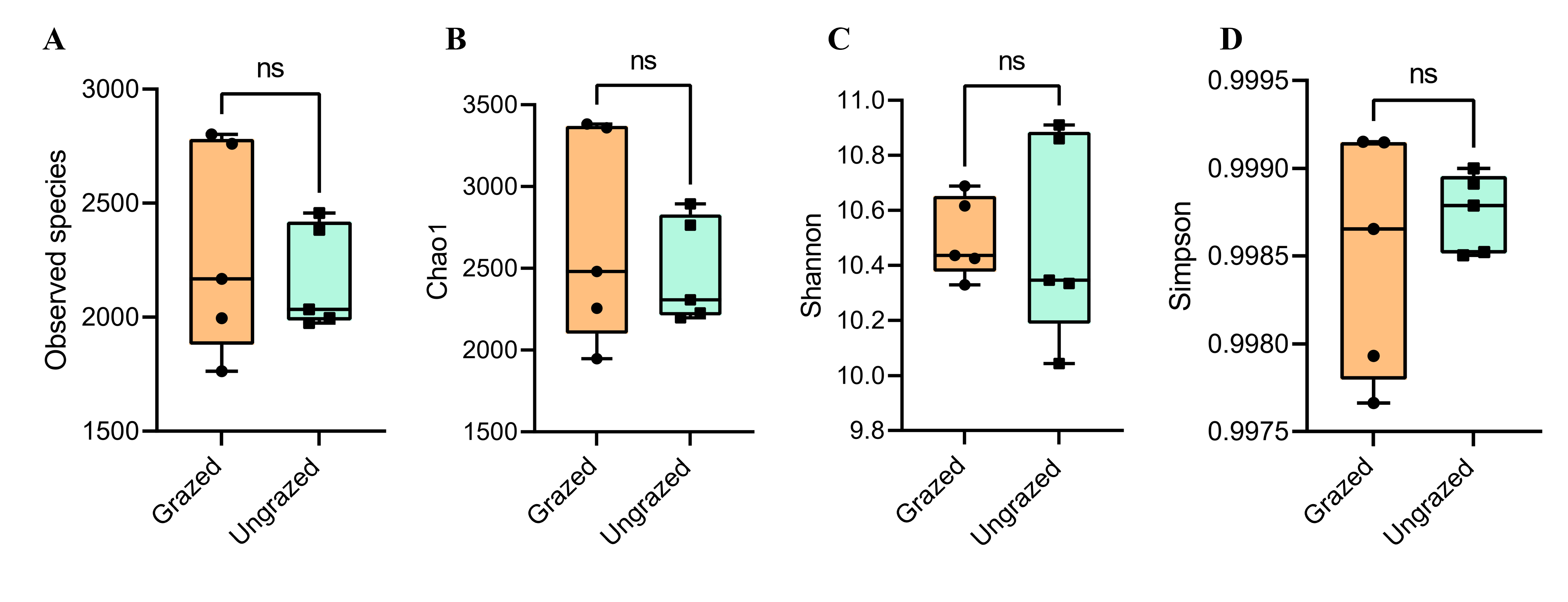

Supplement: Supplemental Information 7 — (A) Observed species, (B) Chao1 index, (C) Shannon index, and (D) Simpson index. Significance was determined using sign test (p < 0.05). [file peerj-12-18433-s007.png]
